# Preferences of Iranian medical students for selecting the compulsory service plan packages: A discrete choice experiment

**DOI:** 10.1002/hsr2.2213

**Published:** 2024-06-25

**Authors:** Enayatollah Homaie Rad, Mohamad Hajizadeh, Mohammad Rajabpour, Zahra Mohtasham‐Amiri, Morteza Rahbar‐Taramsari, Faezeh Bahador, Ehsan Esmaeili Shoja

**Affiliations:** ^1^ Social Determinants of Health Research Center, Trauma Institute Guilan University of Medical Sciences Rasht Iran; ^2^ Canada Research Chair in Health Economics, School of Health Administration, Faculty of Health Dalhousie University Halifax Canada; ^3^ Guilan Road Trauma Research Center, Trauma Institute Guilan University of Medical Sciences Rasht Iran; ^4^ Unit of International affairs Shahid Beheshti University of Medical Sciences Tehran Iran

**Keywords:** compulsory plan, discrete choice experiment, Iran, medical students, preferences

## Abstract

**Background and Aims:**

Health policymakers face challenges in designing compulsory plan packages for medical students to encourage them to work in disadvantaged regions. Using a discrete choice experiment, this study assessed the preferences of medical students for selecting the compulsory service plan packages in Guilan Province, Iran.

**Methods:**

In total, 374 medical students responded to a survey inquiring about salary, distance from their residency city, availability of welfare amenities, work difficulty, the developmental status of their workplace, contract duration, and preference for urban or rural work settings.

**Results:**

The study revealed that students favor a compulsory service package that provides higher salaries and shorter contract duration. They also show a preference for working within their home province over other factors. For the opportunity to serve in their city of residence, they would forgo an average of US$77.93 per month.

**Conclusion:**

While financial incentives were the primary consideration for medical students when choosing compulsory service packages, a range of nonfinancial factors significantly influenced their decisions as well.

## 1 INTRODUCTION

The geographical distribution of general physicians (GPs) is an important factor in promoting the accessibility of healthcare services.^1^, ^2^ According to the World Health Organization (WHO), several countries are faced with critical labor shortages, and more than millions of health workers are needed to fill the gap caused by health human resources shortages in the world.^3^ Approximately 60 million medical personnel were distributed unequally in the last decade, mostly favoring urban areas.^4^, ^5^ For example, more than 96%–97% of medical graduates work in urban areas in Iran.^6^ This unequal distribution of physicians between urban and rural areas is related to the fact that resident physicians practicing in urban areas earn more income, have more opportunities for promotion, have better access to infrastructures, and have better social adaptation than those practicing in rural areas.^4^

A considerable segment of Iran's population resides in areas that are economically underprivileged and lack adequate resources.^2^ To enhance healthcare accessibility in underserved regions, Iran's Ministry of Health and Medical Education (MoHME)—the primary organization for healthcare provision—has instituted a compulsory service program requiring GPs to serve in these areas upon graduation from medical school.^7^, ^8^ In Iran, medical graduates are required to serve 2 years for the government before they can utilize their medical credentials. These compulsory services may be fulfilled in various healthcare settings, including hospitals, public health services, and both rural and urban areas, across all medical disciplines. Compensation varies according to the nature of the work.^9^, ^10^ GPs in Iran often prefer practicing in urban areas due to the challenging working conditions, low job satisfaction, societal issues, and inadequate security prevalent in some underprivileged regions.^11^

It has been shown that young physicians' choices to practice in underdeveloped areas are shaped by multiple factors, including financial incentives, career, and academic growth opportunities, access to private‐sector employment, and the ability to achieve a work‐life balance.^12^ GPs might be more inclined to serve in rural and underserved areas if they are offered housing, job opportunities,^4^ service‐related loans, supplemental earnings, and other financial incentives.^13^, ^14^

While previous research has identified factors that motivate younger general practitioners to work in rural settings, there is scant evidence regarding the specific preferences of Iranian medical graduates that could encourage their practice in underdeveloped areas.^15^, ^16^ Additionally, no studies have been found that analyze the attitudes of medical students toward the mandatory service program. Therefore, this study aims to evaluate the preferences of medical students when choosing their work location under the compulsory service programs in Guilan Province, Iran. Specifically, we employed the discrete choice experiment (DCE) technique to model the preferences of medical students. The DCE effectively highlights the relative importance of various factors, providing policymakers with quantitative data to inform decision‐making.^17^, ^18^ Consequently, this enables the design of more appealing compulsory service plans for medical graduates in Iran. For these plans to be effective, health policymakers must be informed by the study's findings, which can guide the development of enhanced service packages for medical students.

## 2 METHODS

### 2.1 Study setting

Guilan Province, located in northern Iran, had a population of 2.5 million in 2021. Characterized by its moderate level of development and high population density, the region's healthcare services are managed by the Ministry of Health and Medical Education (MoHME) in collaboration with the Guilan University of Medical Sciences (GUMS). GUMS contributes to the healthcare workforce by graduating approximately 120 medical students each semester. Due to the appealing climate, the province has drawn a significant number of new residents, particularly from regions affected by drought over the past decade.^19^ We chose Guilan Province as the focus of our study because gaining insights into the preferences of medical graduates can inform the management of healthcare delivery, particularly in response to the demographic changes occurring within this province.

### 2.2 Survey design

In this study, we utilized the DCE to identify the preferences of medical students at the GUMS. Initially, we carried out nine semistructured interviews with two health economists, two GPs, two medical students, a compulsory plan manager, a medical education specialist, and a head of the medical university to find factors influencing students' choices of compulsory service packages. In addition, we conducted a thorough review of the literature to identify factors associated with the selection of these compulsory plan packages.^4^, ^15^, ^20^ Through a focus group discussion panel with three health economists, two medical education specialists, three medical labor market specialists, two head of universities, we identified the top five critical factors influencing the selection of work placements: salary, contract duration, workplace remoteness, work difficulty, and the developmental status of the workplace, along with the availability of welfare amenities and the location of work. The combinations of factors resulted in 6480 possible alternatives (i.e., 5×4×3×3×3×4×3). From these items, using an orthogonal method, 18 choice sets were selected (D‐efficiency = 0.569).^21^ Table 1 shows a sample of the choice sets. The Excel file of 18 choice sets is added in Supporting Information: Appendix 1. Further information about age, gender, household income, and marital status was also included in the survey question. We used *the dcreate* command of STATA to create 18 choices.^21^ Using the modified Fedorov algorithm, the *dcreate* command produces efficient designs for DCE.^17^, ^22^

**Table 1:** An example of choices.

Question	Choice A	Choice B
Salary	200 million IR. Rials	70 million IR.Rials
Workplace remoteness	Nonneighbour province and far from the province of residence	City of residence
Work difficulty	Easy	Difficult
The developmental status of the workplace	Semi‐developed	Less‐developed
The availability of welfare amenities	Home and transportation	Home
Contract duration	24 months	18 months
Location of work	City	Rural area

We considered the price for salary as a continuous variable in the final model. As per Bliemer et al.,^18^ the minimum number of choice sets is calculated as: $S\geq \frac{k}{j-1}$, where k is the total number of parameters (based on Table 4, k = 16) and j is the number of alternatives (*j* = 2). Thus, the minimum number of choice sets equals 16, which we utilized 18 choices.

### 2.3 Data collection and interviews

The telephone number of students who had passed their comprehensive exam and had started the internship courses in hospitals was gathered from the Deputy of Education of the GUMS. In Iran, the duration of medical education is 7 years. Students take a comprehensive exam at the end of their fifth year, followed by a minimum 2‐year internship program in a hospital.^23^, ^24^, ^25^

The eligible students were contacted by a researcher to provide a comprehensive explanation of the aim and the instructions of the survey. Text messages were sent to those who were given written consent to participate in the survey. The text message contained an address link to the online questionnaire. We used *the Porsline* tool (https://survey.porsline.ir/s/OftzPRz/) to conduct the survey online. The *Porsline* is a local tool that provides effective designs for DCE. Written informed consent forms were obtained from the participants before starting the interview. We ensured that all of the participants were aware of the confidentiality of their information. The number of students who had the eligibility to be included in the survey was 533. Of these students, 48 (9%) did not respond to the calls or we could not access their phone numbers, and 83 (15.6%) did not accept to participate in the survey. This resulted in a total of 402 students who completed the survey (response rate: 75.4%). By analyzing the time of answering the survey questions, we deleted data of the respondents who answered the questions very fast (less than 1.5 min) and had a wrong answer to an unambiguously better alternative. This yielded a final sample of 374 students who were still students at the time of the survey. The survey was designed in Persian language and the participants were obliged to select between each of the choice sets. The study was approved ethically by the deputy of research, GUMS (Ethics code: IR.GUMS.REC.1399.108).

### 2.4 Statistical analysis

The random utility theory is the theoretical background of the DCE.^26^ Based on the random utility, each person selects a choice if and only if the utility of that choice is higher than its alternatives. For example, if a student chooses a higher versus less developed workplace, this implies that a higher developed region offers more utility. In our study, we compared different alternatives to the compulsory plan in a mixed logistic regression model as follows:

(1) $$U_{i}=\beta_{1}+\beta_{2}.{\mathrm{pay}}_{i}+\beta_{3}.{\mathrm{leng}}_{i}+\beta_{4}.{\mathrm{loc}}_{i}+\beta_{5}.{\mathrm{welf}}_{i}+\beta_{6}.{\mathrm{urb}}_{i}+\beta_{5}.{\mathrm{dev}}_{i}+\beta_{5}.{\mathrm{hard}}_{i}+\varepsilon_{i},$$

where $U_{i}$ is the utility of alternative i. The dependent variable of the model, U is a subjective variable that cannot be calculated directly but can be compared between alternatives. The definitions of independent variables and their levels are provided below.

pay denotes the monthly salary for the compulsory plan for the physician. As a rational choice, people like to earn more money, and higher salaries produce more utility. This variable was asked in the local currency (IR.Rials) and converted into US$ (Exchange rate: R.Rials185,000 = US$1). This variable had five levels US$217, US$379, US$541, US$811, and US$1081. The highest level (US$1081) is approximately 25% higher than the highest amount of current monthly salary of physicians and the lowest one (US$217) is the minimum wage rate of Iran in 2019.^27^

leng indicates the contract duration in months in a compulsory plan. As a rational choice, GPs like to end the compulsory plan earlier because there several better opportunities for the physicians outside of the compulsory plan in both public and private sectors. In other words, the lower length of the plan has more utility for the GPs. This variable had four levels of 14, 16, 18, and 24 months, of which 14 is the lowest length of the plan due to Iranian compulsory laws and 24 is the highest.

loc indexes the workplace remoteness. The levels of this variable contain the city of residence, the province of residence, the neighbor province to the province of residence, non‐neighbor province but near to the province of residence, non‐neighbor province, and far from the province of residence. We defined the city of residence as the city or town in which the students and his/her parents lived before they started their medical program. Medical graduates might prefer to work near their place of residence, but this selection cannot be generalizable to all graduates and is highly related to the culture, residence location condition, and family loyalty.

welf is the availability of welfare amenities besides the salary for the compulsory plan for the physician. This variable had four levels nothing, transportation facilities, home, home, and transportation. As a rational selection, the physician prefers more welfare amenities.

urb indicates the location of work in urban or rural regions. Some physicians might like to work in urban regions and others might like to work in rural regions.

dev is the development status of the workplace. Physicians generally like to work in higher‐developed regions. This variable had three levels highly developed, semi‐developed, and less‐developed.

dif shows the work difficulty. We defined this variable as how much the work is crowded, complicated, stressful, and needs emergency conditions. In a rational setting, the physician likes to select easier works than harder ones. This variable had three levels easy, average, and difficult.

Willingness to pay (WTP) was calculated as follows:

(2) $$\mathrm{WTP}\;\left(\mathrm{leng}\right)=-\frac{\sigma U/\sigma \mathrm{leng}}{\sigma U/\sigma \mathrm{pay}}=-\frac{\beta_{3}}{\beta_{2}},$$

where $\beta_{3}$and $\beta_{2}$ are the coefficients obtained from Equation 1. The pay and leng variables were not distributed normally. Thus, if we utilized the mixed effect logistic regression model, WTP results might not be estimated correctly. To avoid this bias in calculating WTP for each attribute, we estimated the model using a Bayesian mixed logistic estimator, which is considered to be the best in the DCE studies.^28^ Salary variables were added in both continuous and dummy forms and other attributes were added in dummy form. The number of Halton draws used in the estimation was 2000 and the number of burns was 1000.

To test the validity of the experiment, we added a rational choice set. The wrong answer to the rational choice set meant that the participant did not notice the questions or did not have a rational selection. Twenty‐one of the participants selected the unambiguously better alternative. Furthermore, we divided the data into two groups of males (*n* = 149) and females (*n* = 225), and low‐income (*n* = 141) and high‐income groups (*n* = 259) and reanalyzed the model using the Bayesian mixed logistic estimator. A self‐assessed Likert‐scale question ranging from 0 to 10 (visual analog scale technique) was added to the study. We categorized the participants into low and high‐income groups at the cut‐off point of 5. A score below 5 classified a participant as belonging to the low‐income group, while a score above 5 indicated the high‐income group. The *p* values under 0.05 were considered as significant. All estimations were performed using STATA SE software V. 14.1. To calculate WTP, we used the *nlcom* command in STATA.

## 3 RESULTS

Table 2 shows descriptive statistics of the sample. As shown in the table, the mean age of participants was 26.25 ± 0.15 years. The mean household monthly income was US$405.62 ± US$7.39. Out of 317 participants, 225 (60.16%) were females, 57 (15.24%) were married, 163 (43.58%) were residents of Guilan Province, and 6 (1.6%) lived in rural regions.

**Table 2:** Descriptive statistics of the sample.

Variable	Frequency (Percentage)	Variable	Frequency (percentage)
Residency		Marital status	
Urban	368 (98.4)	Single	317 (84.76)
Rural	6 (1.6)	Married	57 (15.24)
Province of residence		Gender	
Other provinces	211 (56.42)	Female	225 (60.16)
Guilan	163 (43.58)	Male	149 (39.84)

**Variable**	**Mean (Standard deviation)**	**Variable**	**Mean (Standard deviation)**
Age	26.25 (0.15)	Household income	405.62 (7.39)

**Figure 1:**
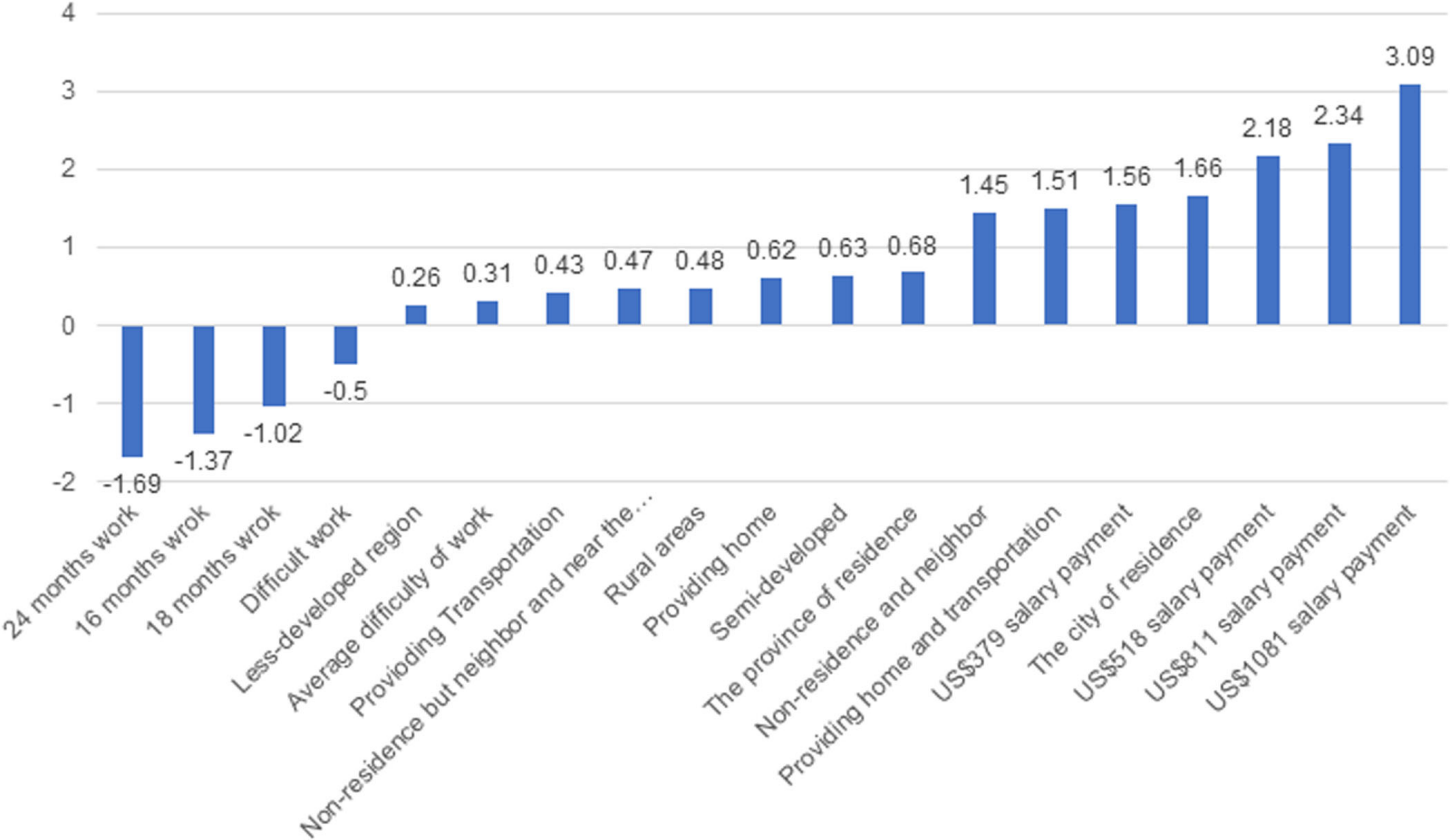
Preferences rank of attributes used in the study.

Table 3 shows the results of Bayesian mixed logistic regression. As reported in the table, physicians prefer the highest amount of payment, that is, US$1081 (coefficient = 3.09, 95% confidence interval [CI]: 3.01–3.16), US$811 (coefficient = 2.34, 95% CI: 2.27–2.42) work at the city of residence (coefficient = 1.66, 95% CI: 1.58–1.74), work at nonresidence and neighbor (coefficient = 1.45, 95% CI: 1.39–1.51), semi‐developed region than less developed one (coefficient = 0.63, 95% CI: 0.56–0.7) and both home and transportation services (coefficient = 1.51, 95% CI: 1.45–1.57) more than other attributes. Figure 1 displays the ranking of attributes according to preference. The attributes are ordered from most to least preferred, as depicted in the figure. The use of dummy variables allows for the comparison of all attribute coefficients with one another.

**Table 3:** Bayesian mixed logit estimator to estimate preferences ranks of all variables.

Variable	Levels	Coefficient	Standard error	*p* Value	95% confidence interval
Salary	US$217	Ref			
	US$379	1.56	0.04	<0.001	1.48 to 1.63
	US$518	2.18	0.03	<0.001	2.12 to 2.25
	US$811	2.34	0.04	<0.001	2.27 to 2.42
	US$1081	3.09	0.04	<0.001	3.01 to 3.16
Contract duration	14 months	Ref			
	16 months	−1.37	0.03	<0.001	−1.43 to −1.31
	18 months	−1.02	0.03	<0.001	−1.08 to −0.95
	24 months	−1.69	0.03	<0.001	−1.76 to −1.63
Workplace remoteness	Nonresidence and far	Ref			
	Nonresidence but neighbor and near	0.47	0.03	<0.001	0.4 to 0.54
	Nonresidence and neighbor	1.45	0.03	<0.001	1.39 to 1.51
	The province of residence	0.68	0.03	<0.001	0.62 to 0.75
	The city of residence	1.66	0.04	<0.001	1.58 to 1.74
Work difficulty	Easy	Ref			
	Average	0.31	0.03	<0.001	0.25 to 0.38
	Difficult	−0.50	0.03	<0.001	−0.56 to −0.43
Development status of the workplace	High‐developed	Ref			
	Semi‐developed	0.63	0.04	<0.001	0.56 to 0.7
	Less‐developed	0.26	0.03	<0.001	0.21 to 0.32
Welfare amenities	None	Ref			
	Transportation	0.43	0.03	<0.001	0.37 to 0.49
	Home	0.62	0.04	<0.001	0.53 to 0.7
	Home and transportation	1.51	0.03	<0.001	1.45 to 1.57
Location of work	City	Ref			
	Rural areas	0.48	0.04	<0.001	0.41 to 0.56

*Note*: Ref indicates reference category in the regression.

Table 4 shows the results of WTP for compulsory plan services among medical students. The findings indicated that students prefer to be compensated an additional US$95.31 monthly during their 24‐month compulsory service. The willingness to pay (WTP) for this increase is ‐US$95.31 (95% CI: −113.88 to −76.73). The WTP was ‐US$ 33.48 (95% CI: −52.30 to −14.66) for 18 months compulsory plan and US$14.81 (95% CI: −1.97 to 31.59) for 16 months compulsory plan. Furthermore, students showed a WTP of US$77.93 (95% CI: 59.85–96.01) for a compulsory service plan located in a city of residence. For positions in a province of their home province, the WTP was US$35.78 (95% CI: 17.23–54.33). The WTP for positions in neighboring provinces is not statistically significant at the 95% confidence level. The WTP for working in a province that is neither the place of residence nor adjacent but still close to it was ‐US$50.05 (95% CI: −65.19 to −34.92). The negative WTP values suggested that medical students would prefer additional compensation for work locations situated further from their place of residence. For a workplace with average difficulty, the WTP was not statistically significant at the 95% confidence level. In contrast, for difficult work conditions, the WTP was ‐US$84.57 (95% CI: −104.06 to −65.09). The WTPs for working in less‐developed and semi‐developed regions were ‐US$64.80 (95% CI:−85.31 to −44.30) and US$100.73 (95% CI: 80.012 to 121.34), respectively. The WTP for working in a rural area was ‐US$49.55 (95% CI: −68.26 to −30.84). The WTP to forego transportation services was ‐US$53.05 (95% CI: −73.11 to −32.99). The WTP for housing provision was not statistically significant. Finally, WTP of both housing and transportation services was US$24.41 (95% CI: 5.18–43.64).

**Table 4:** Willingness to pay (WTP) for the compulsory plan among medical students of Iran.

Variable	Choice	WTP (US$)	Standard Error	*p* Value	95% confidence interval
Salary	Payment in US$	0.06			
Contract duration	14 months				
	16 months	14.81	8.51	0.083	−1.97 to 31.59
	18 months	−33.48	9.54	0.001	−52.30 to −14.66
	24 months	−95.31	9.42	<0.001	−113.88 to −76.73
Workplace remoteness	Nonresidence and far				
	Nonresidence but neighbor and near	−50.05	7.68	<0.001	−65.19 to −34.92
	Nonresidence and neighbor	−6.42	6.65	0.335	−19.54 to 6.69
	The province of residence	35.78	9.41	<0.001	17.23 to 54.33
	The city of residence	77.93	9.17	<0.001	59.85 to 96.01
Work difficulty	Easy				
	Average	−14.24	8.03	0.078	−30.08 to 1.60
	Difficult	−84.57	9.88	<0.001	−104.06 to −65.09
Development status of the workplace	High‐developed				
	Semi‐developed	100.73	10.45	<0.001	80.12 to 121.34
	Less‐developed	−64.80	10.40	<0.001	−85.31 to −44.30
Welfare amenities	None				
	Transportation	−53.05	10.17	<0.001	−73.11 to −32.99
	Home	11.63	7.15	0.105	−2.46 to 25.72
	Home and transportation	24.41	9.75	0.013	5.18 to 43.64
Location of work	City				
	Rural areas	−49.55	9.49	<0.001	−68.26 to −30.84

Table 5 shows the results of Bayesian mixed logistic regression by sex and income level. As female students may be more dependent on their families and may not like to work far from their city of residence, we separated our analysis by sex. In addition, since earning money may not be an important issue for the higher‐income group compared with the lower‐income group, we performed our analysis for high‐income and low‐income groups, separately using the self‐assessed Likert scale financial status. In addition, we considered the salary payment variable as a fixed variable in the model. As reported in the table, the preferences of males and females were similar; however, the coefficients of the model were higher in females than males, which can indicate that females make more value for their selection. For instance, the preference for working in their city of residence is represented by a coefficient of 46.25 for males compared with 82.33 for females. Similarly, the coefficient indicating the preference to work in their province of residence is 5.83 for males and 23.26 for females. In addition, the coefficient for working in difficult conditions was −82.80 for females, while it was −17.78 for males. As shown in Table 5, both high‐income and low‐income groups prefer the lower length of a compulsory plan. However, the smaller magnitude of the low‐income group coefficient compared with the higher group indicates that they like to complete their plan earlier. In addition, working in the residence city has more value for the low‐income group. Home and transport are more important for the low‐income group than their high‐income group counterpart (coefficient of lower income group = 39.96 vs. the higher income group = 18.32). Although the coefficient on salary payment was similar for the male and female groups (0.05), it was higher for the low‐income group than the high‐income group (0.04 vs. 0.06).

**Table 5:** Bayesian mixed logit estimations of students' preference to select the compulsory plan by gender and household income.

Variable	State	Total population		Males		Females		Low‐income group		High‐income group	
		Coefficient	Standard error	Coefficient	Standard error	Coefficient	Standard error	Coefficient	Standard error	Coefficient	Standard error
Salary	Payment in US$	0.05**	0.02	0.05**	0.01	0.05**	0.01	0.06**	0.02	0.04**	0.02
Contract duration	14 months										
	16 months	14.31**	6.92	24.79**	5.15	16.86	10.62	20.00**	9.47	24.93**	7.23
	18 months	−56.01**	8.44	21.31**	5.50	−47.18**	9.90	−23.94	15.44	20.43**	7.15
	24 months	−73.90**	9.54	−37.33**	6.29	−71.55**	12.25	−90.81**	13.72	−16.61**	6.49
Workplace remoteness	Nonresidence and far										
	Nonresidence but neighbour and near	−15.43**	6.66	−40.33**	6.70	−16.38	9.95	−49.74**	10.40	−8.04	4.92
	Nonresidence and neighbor	−15.05**	6.47	12.15**	6.67	−13.38	10.69	−10.06	9.06	24.90**	8.50
	The province of residence	33.27**	7.26	5.83	4.40	23.26*	8.74	10.11	9.02	12.16*	6.66
	The city of residence	64.10**	8.63	46.25**	6.62	82.33*	15.78	64.08**	11.80	42.35**	8.39
Work difficulty	Easy										
	Average	−24.09**	7.28	−6.46	6.22	−25.39*	12.39	−49.02**	11.90	11.25**	5.64
	Difficult	−74.33**	9.61	−17.78**	5.20	−82.80*	12.96	−74.97**	12.69	−7.00	6.40
Development status of the workplace	High‐developed										
	Semi‐developed	67.88**	7.89	44.86**	7.50	64.29*	12.05	71.46**	17.12	54.31**	11.86
	Less‐developed	−15.86**	6.76	−32.50**	6.63	−25.92*	10.05	−46.99**	13.11	2.88	4.41
Welfare amenities	None										
	Transportation	−43.07**	9.08	−11.83*	6.09	−30.25*	11.23	−39.73**	12.97	−3.84	4.38
	Home	15.18**	7.10	6.84	5.77	12.02	10.50	1.29	9.75	21.75**	6.32
	Home and transportation	29.96**	6.55	12.97*	6.71	27.04*	9.35	39.96**	11.89	18.32**	5.63
Location of work	City										
	Rural areas	−51.48**	8.46	−20.36**	6.58	−41.17*	9.19	−60.89**	12.70	−11.38*	5.87

*** *p* Value < 0.01

** *p* Value < 0.05

* *p* Value < 0.1.

## 4 DISCUSSION

This study indicated income as the most important factor affecting the utility of compulsory plans to physicians. This finding was similar to the results of previous studies conducted in Germany,^29^ the UK, Cameroon, and Ghana.^14^, ^20^, ^30^ It is not surprising that financial benefit plays an important role in selecting the service package among Iranian medical students, especially among low‐income groups. The effect of wealth and income on WTP and utility was confirmed in some recent studies.^31^, ^32^, ^33^ The value for payments was similar for males and females, which was confirmed in another study.^14^

The selection of the package is not related to the payment only. Students prefer lower contract duration, working in place of residence, and greater welfare amenities. They do not prefer to work in less‐developed regions, difficult work conditions, and rural areas. A recent systematic review found that nonfinancial factors are as important as financial factors in selecting deprived areas for working by physicians in Iran.^11^ Findings showed that the participants like to work in semi‐developed regions and like to earn less money in this condition. This might arise from the level of development of the country. Iran is a developing country and working in semi‐developed regions is not unfavorable for Iranians.^34^ Results showed that Iranian medical students prefer to do their compulsory plan in their city of residence, and females compared with males have more desire to work in their city of residence. This may be related to cultural factors and very strong loyalty among Iranian families, which encourages children to live near their parents' city.^35^, ^36^, ^37^ In other words, the higher preference among females to work in the city of residence can also be explained by their higher loyalty to their families and the quality of their relationship with family compared with males.^38^, ^39^ The results of a DCE study suggested that medical students in Iran do not prefer to work in provinces far from their province of residence.^15^ In contrast to our finding, a study in Ghana found that neither financial nor nonfinancial incentives change the willingness of medical students to work in rural areas.^40^

The results of this study showed that medical students like to receive an additional US$49.55 per month for working in rural areas and US$64.8 for working in less‐developed areas. In a study conducted in Cameroon, the WTP medical students working in urban areas was US$116.2, which was higher than the present study.^30^ Another study showed that physicians in England accept working in deprived areas for an additional £5000 annually.^41^ A study in Sudan found that junior physicians do not prefer rural regions.^42^ Similar to findings from a study in China, which demonstrated a preference among medical students for working in urban settings, our research also revealed that female students have a stronger preference for urban areas compared with their male counterparts, aligning with the Chinese study.^43^ Another study in three countries of Pakistan, Turkey, and Spain showed that medical students like to work in desirable locations.^44^ As a rational finding, medical students gain less utility from difficult work, while the utility loss of difficult work for females is greater than for males. A recent study in Iran showed that females do not work in professions such as orthopedics, emergency medicine, and cardiology.^27^ A study in Pakistan showed that work conditions as the most important factor for specialty training positions.^44^ The results also suggested that medical students like to pay monthly US$24.41 for home and transportation benefits in the compulsory plan, respectively. These results were similar to two previous studies in Iran and Sudan.^15^, ^42^ The average monthly rent for a 45 m^2^ house was US$45 in Iran in 2020.^45^ Medical students like to pay less than the average rent price for this service. In addition, the coefficient on home and transportation was greater in female than male students. This shows the importance of secure welfare amenities for females. In contrast to our finding, a study in Ghana showed that males valued housing more than females, but females preferred the utility of cars.^14^

The contract duration was another important factor that changes the utility of compulsory plans to medical students. Based on the results of our study, medical students prefer shorter compulsory plans. The 16‐month compulsory plan was the most favorable plan for medical students, and they do not prefer 18 and 24‐month compulsory plans. This can be explained by students having better work conditions and can earn more money in the private sector after completing their compulsory plan.^46^ Two different DCE studies in Ghana and Sudan found that medical students favor shorter contracts.^14^, ^42^ In addition, the length of the compulsory plan was more important for the low‐income group than the high‐income group. The low‐income group prefers to end the compulsory plan earlier than the high‐income group because the utility of earning additional money is greater for the lower compared with the higher‐income group, that is, the law of diminishing marginal utility of income.^46^, ^47^, ^48^

The findings of this study can help health policymakers find better strategies for decreasing the geographical inequity of physicians in Iran. The MoHME must find those places which are faced with a shortage of physicians. These places are mainly located in less‐developed regions, rural areas, and difficult work positions. To encourage physicians to work in these areas, payments must be flexible and related to the preferences and WTP of physicians. Studies have shown that physicians are not satisfied with the compulsory plan.^49^, ^50^, ^51^, ^52^ Nevertheless, evidence has shown that these programs can be helpful and effective. For example, the physician shortages program in the United States successfully increased retention rates in rural areas and improved equity in healthcare.^53^, ^54^, ^55^ Understanding the preferences of physicians can improve the job satisfaction of physicians. The payments can be calculated by adding each attribute and asking physicians to select their favorite attribute. Then, the payments can appear for the physicians and if the GPs are satisfied with the payment, they can select the package. By increasing in the payments of GPs, the health policymakers can encourage them to select those attributes which are less‐preferred to be selected (like lower developed places, harder works, etc.)

This study was subject to some limitations. First, we assessed the preferences of the medical students studying the GUMS; thus, the result may not be generalizable to all Iranian medical students. DCE assumes that the responses reflect real‐life choices, while the real choices might be different. Second, while additional attributes such as the permission to work in the private sector, employment in public health and medical services, and roles in research could have been considered for the study, they were not included due to constraints on the number of attributes we could examine and the complexity they would add to the questionnaire. Third, in our subgroup analysis, certain coefficients were not statistically significant at a 95% confidence interval. This lack of significance could be attributed to smaller sample sizes within some subgroups or potential questions concerning the study's validity. Alternatively, it may indicate a level of indifference among participants when it comes to selecting attributes pertinent to that specific choice. Fourth, there are some guidelines like the WHO guideline for conducting DCE studies which we did not use in the present study.^56^, ^57^ Fifth, the WTP for transportation services was unexpected. WTP of transportation services was negative which meant that the participants preffered to earn more mony in the condition they have access to transportation services. As the utility theory, people like to pay for those conditions which had positive benefit to them. It might be related to the level selection of study variables. however in the rank of preferences (Table 3) the transportation coefficient was positive which indicated that the participants preferred using transportation services. Sixth, the interpretation of development as an attribute for selecting a place for compulsory services might be different for the students. Whoever, Before the interview, we facilitated a discussion with the students about regional development, using a survey for guidance. We asked the participants to think about their socioeconomic status due to the discussion and categorize the development by their interpretation.

## 5 CONCLUSION

Financial incentives remain the most important factors that affect medical students' preferences for selecting compulsory plan packages. Nonfinancial incentives also affect the preferences of the medical student. To improve equity in the distribution of physicians in economically disadvantaged areas and job satisfaction of GPs in Iran, health policymakers must consider the preferences of medical students in designing compulsory packages.

## AUTHOR CONTRIBUTIONS

**Enayatollah Homaie Rad**: Conceptualization; methodology; software; supervision; writing—review and editing. **Mohamad Hajizadeh**: Writing—original draft; writing—review and editing; formal analysis. **Mohammad Rajabpour**: Data curation; validation; writing—review and editing. **Zahra Mohtasham‐Amiri**: Writing—review and editing; data curation. **Morteza Rahbar‐Taramsari**: Supervision; validation; writing—review and editing. **Faezeh Bahador**: Resources; formal analysis; writing—review and editing. **Ehsan Esmaeili Shoja**: Data curation; software; writing—review and editing.

## CONFLICT OF INTEREST STATEMENT

The authors declare no conflict of interest.

## TRANSPARENCY STATEMENT

The lead author Enayatollah Homaie Rad affirms that this manuscript is an honest, accurate, and transparent account of the study being reported; that no important aspects of the study have been omitted; and that any discrepancies from the study as planned (and, if relevant, registered) have been explained.

## Supporting information

Supporting information.

## Data Availability

The authors confirm that the data supporting the findings of this study are available within the article's supplementary materials. All authors have read and approved the final version of the manuscript. The corresponding author had full access to all of the data in this study and took complete responsibility for the integrity of the data and the accuracy of the data analysis.
